# Meeting of the New York State Medical Society

**Published:** 1876-06

**Authors:** 


### Meeting of the New York State Medical Society.

The annual meeting of the Medical Society of the State of New York, was held in Albany, Tuesday, June 20th, Dr. Rochester, of this city, presiding. We present elswhere the President's inaugural address, which will be found to be an able and interesting review of the prominent medical questions of the day. The following is the list of officers elected:

*President*—Dr. E. R. Squibb, of Brooklyn.
*Vice-President*—Dr. J. V. Kendall, of Baldwinsville.
*Secretary*—Dr. Edward R. Hun, of Albany.
*Treasurer*—Dr. Charles H. Porter, of Albany.
*Censors*—Southern District, E. R. Peaslee, New York; E. H. Parker, Poughkeepsie; Ellsworth Eliot, New York. Eastern District, Henry B. Whiton, Troy; Jas. L. Babcock, Albany; John P. Sharer, Little Falls. Middle District, M. M. Bagg, Utica; Geo. W. Cooke, Otego; C. G. Bacon, Fulton. Western District, C. C. Wyckoff, Buffalo; Harvey Jewett, Canandaigua; Caleb Green, Homer.

*Committee on Prize Essay*—Henry W. Dean, Wm. S. Ely, Monroe; Julius F. Miner, Erie.

*Committee on Publication*—E. R. Hun, Chas. H. Porter, Wm. H. Bailey, Albany.

*Committee on By-Laws*—Wm. C. Wey, Chemung; E. R. Hun, Wm. H. Bailey, Albany.

*Censor of Syracuse University*—B. F. Sherman, St. Lawrence.

